# Evaluating the Study Designs and Outcome Measures Used in Service User Involvement in Health Professional Entry‐Level Education: A Systematic Review

**DOI:** 10.1111/hex.70439

**Published:** 2025-09-27

**Authors:** Jonathan Green, Jennifer Oates, Sam Robertson, Elizabeth Barley, Chris Jacobs

**Affiliations:** ^1^ School of Health Sciences University of Surrey Guildford UK; ^2^ Sussex Partnership NHS Foundation Trust Worthing UK

**Keywords:** allied health professional, healthcare education, midwifery, nursing, outcome measures, pharmacy, service user involvement, social work, undergraduate education

## Abstract

**Background:**

It is a regulatory requirement in the United Kingdom and Australia that people who use services are involved in health professional education. Evaluating service user involvement aims to inform curriculum development and improvement. However, although there is research evaluating service user involvement in medical education, optimal outcome measures for other health professionals have not been identified.

**Objective:**

This study focused on service user involvement in entry‐level education for nurses, midwives, allied health professionals, social workers and pharmacists. The aim was to (i) identify study characteristics, designs and methods used to measure outcomes, (ii) describe the characteristics of outcome measures used and (iii) identify the extent to which the outcome measures aligned with the modified Kirkpatrick Evaluation Framework.

**Search Strategy:**

Medline, CINAHL and PsychINFO databases were systematically searched for studies published over a 24‐year period between 2000 and 2024. Two reviewers independently screened studies. A narrative synthesis was conducted. Measures were mapped to the modified Kirkpatrick Evaluation Framework.

**Results:**

Nineteen studies using 29 measures were selected. Study designs were mostly quasi‐experimental with small university‐based samples. Data were typically collected pre‐ and post‐service user involvement, assessing changes in student knowledge and attitudes. Measures assessed the perspective of students (*n* = 29) and educators (*n* = 1), but not the service users' perspective (*n* = 0). Eight of the measures were validated; four for student health professionals and four for other populations. No measures aligned with the highest levels of the modified Kirkpatrick Evaluation Framework regarding the impact of service user involvement on the health system and patients.

**Conclusion:**

Limitations in the study designs reduced the comparability and generalisability of the identified studies. None of the measures evaluated the impact of service user involvement on the health system or patients. Educators' perspectives on the service users' involvement in the education were limited. Service users' perspectives were absent. To embed a culture of involvement, future research is needed to identify the requirements of outcome measures from the perspective of service users and educators.

**Patient and Public Contribution:**

Co‐author (S.R.), a lead for service user and carer involvement at an NHS Trust, guided the study design, data analysis and manuscript development.

## 1 Introduction and Background

In the United Kingdom, the National Institute for Health Research [1] defines service user involvement in healthcare as an activity carried out ‘with’ or ‘by’ service users, rather than ‘to’ or ‘for’ them. It is an expectation that people with lived experience should contribute at all levels of system design, service delivery, service monitoring and health professional education [2]. Service user involvement can influence the direction of healthcare towards a person‐centred culture, creating genuine partnerships [3] (National Health Service (NHS) England, 2019), aligning with requirements of the World Health Organization to increase person‐centred services [4].

Service user involvement is integral to health professional education. It is a regulatory expectation for professional preparatory programmes within the United Kingdom and Australia (Nursing and Midwifery Council in the UK 2018; [5, 6]). Educational programmes can be utilised to develop a culture of person‐centred care [7] and provide the earliest opportunity in health professionals' careers to value service user involvement. Health Education England [8] and NHS England (2016) emphasise the central role of service users in health professional education. Evaluating service user involvement as a pedagogical approach can drive curriculum development and improvement [9]. Service user involvement in education encompasses role play, recruitment and selection of students, teaching in the classroom, assessment and feedback, development of learning tools and approaches and curriculum development [10]. Studies have reported on the benefits of service user involvement in health and social care professional entry‐level education for developing student empathy, improving communication and negotiation skills, promoting partnership working, bridging the gap between theory and practice, and challenging preconceived ideas and prejudices [10, 11, 12, 13]. The experience has been described as ‘transformative’, enabling students to gain insight into the service user perspective [14]. Benefits for service users include increased confidence and sense of belonging [15], developing new skills, empowerment and increasing sense of well‐being [10]. However, not all outcomes will be positive. There is a possibility that service user involvement initiatives may fail to drive meaningful change or negatively impact service users' health [16].

There are growing expectations that service users should be involved in health professional education. However, despite there being research evaluating service user involvement in medical education [17], there is little research regarding optimal outcome measures of service user involvement for other health professionals [18, 19] and no agreement on what constitutes the ‘Gold Standard’ of outcome measurement. Without reliable methods to evaluate the effects of service user involvement, the ability to refine educational approaches, justify resource allocation or ensure accountability to both students and service users is limited. Identifying and evaluating outcome measures is essential to realising the values that drive service user involvement. Establishing how best to evaluate service user involvement is therefore essential to advancing both its pedagogical and ethical case, ensuring that service users are not included in a symbolic or tokenistic way, but in ways that are demonstrably impactful. The first step in addressing this gap is to establish what has been measured in reported studies that evaluate service user involvement and to determine the validity and reliability of the methods and instruments used.

The aim of this systematic review was to describe the study designs and outcome measures used to evaluate service user involvement in nursing, midwifery, allied health professionals, social work and pharmacy (non‐medical health professionals) entry‐level education. The objectives were: (i) to identify the study characteristics, designs and methods used to measure outcomes, (ii) to describe the characteristics of the outcome measures used and (iii) to identify the extent to which the identified outcome measures meet the criteria set out in a widely used educational evaluation framework [20]. Our search of the Cochrane Library [21] and PROSPERO database identified no previous or ongoing scoping or systematic reviews in this area.

### 1.1 Evaluation Framework

**Figure 1:**
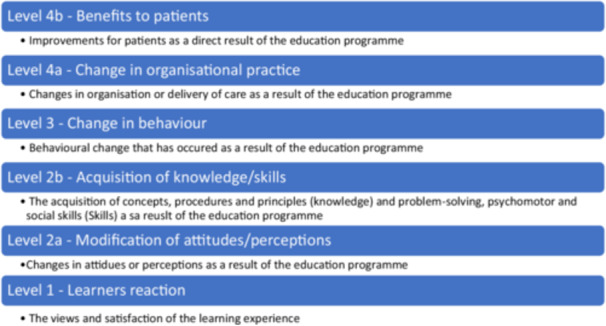
Modified Kirkpatrick's framework of evaluation (adapted from [20]).

Evaluation is essential in health professional education. It enables educators to identify effective pedagogical approaches and forms an important step in the development of teaching initiatives. The Kirkpatrick framework has been frequently used to evaluate the effectiveness of educational service user involvement [22]. The framework evaluates service user involvement across four levels: (1) student reaction to training, (2) student learning, (3) changes in student behaviour and (4) whether the training is achieving results, with each level providing a different perspective on the impact of the training. Barr et al. [20] modified the framework (see Figure 1) by separating ‘student learning’ into ‘modification of attitudes/perceptions’ and ‘acquisition of knowledge/skills’ and ‘results’ into ‘change in organisational practice’ and ‘benefit to patients’. The revised framework identifies that the ultimate purpose is to benefit the service user, and any comprehensive evaluation should identify the impact on service users. We used the modified Kirkpatrick framework to identify how outcome measures evaluate service user involvement in non‐medical health professionals' entry‐level education.

## 2 Methods

This systematic review adheres to the Preferred Reporting Items for Systematic Review and Meta‐Analyses guidelines (PRISMA‐S) [23]. The protocol was registered on PROSPERO available at https://www.crd.york.ac.uk/prospero (ID: CRD42022375920).

### 2.1 Eligibility Criteria

Predefined eligibility criteria were applied to enable systematic identification of articles relevant to the research question (available in the Supporting Information). Studies reporting quantitative methods to measure the outcome of service user involvement in non‐medical health professionals' entry‐level education in higher education institutes were eligible for inclusion in this review. The roles for allied health professionals were defined by the National Health Service England [24, 25] list. Studies were included if they were written in the English language and published in a peer‐reviewed journal between January 2000 and August 2024.

### 2.2 Search Strategy

A search for relevant studies was conducted using the Medline, CINAHL and PsycInfo databases on the EBSCOhost search platform. Pilot searches were conducted on all platforms with the support of a librarian with expertise in conducting literature searches to help refine the search strategy. The Population, Intervention, Comparison, Outcome (PICO) criteria were applied as recommended by the Centre for Reviews and Dissemination [26] to maintain the focus of the search on the research question. MESH terms and Boolean operators were used to link the search terms. Due to the variation in the language used for service user involvement in studies of the provision of education [27], a combination of keywords and controlled vocabulary informed the search terms. The final search was conducted across all platforms on 9 August 2024.

### 2.3 Study Selection

**Figure 2:**
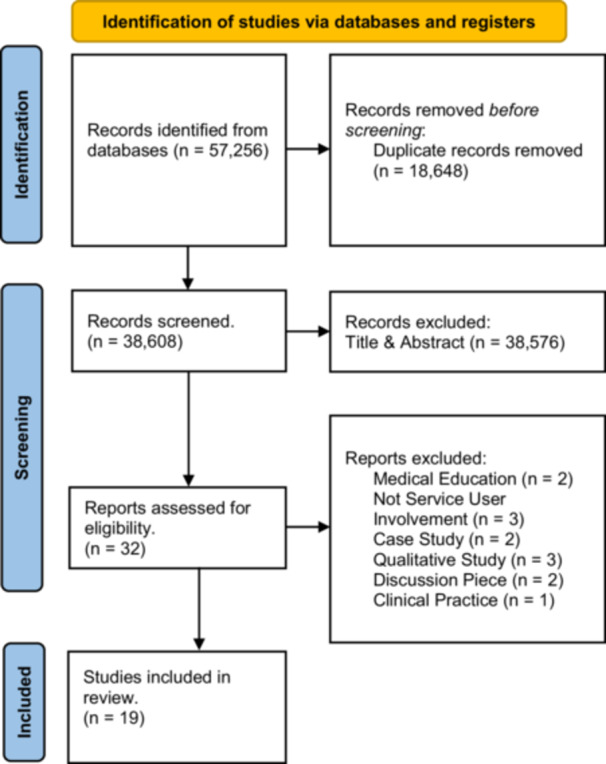
PRISMA diagram.

After deduplication, one reviewer (J.G.) screened all articles by title and abstract based on the inclusion/exclusion criteria. A second reviewer (C.J.) independently screened 20% of titles and abstracts and 20% of selected full‐text articles until good agreement was reached (Cohen's *κ* = 0.73 and 1.0, respectively) (see Figure 2).

### 2.4 Data Extraction

A data extraction form was developed by J.G. and piloted by J.G. and C.J. on 16% (*n* = 3) of the full‐text articles. Data extraction was carried out by J.G. and validated by C.J. When the study did not provide data on the psychometric properties of a measure, the cited source for the measure's reliability and validation study was used.

## 3 Data Analysis

### 3.1 ROB

A risk of bias (ROB) assessment was conducted by J.G. to provide insight into the quality of the articles, rather than as a threshold for inclusion in the review. ROB was assessed by different validated tools depending on the methods utilised, including the Mixed Methods Appraisal Tool (MMAT) [28], the Joanna Briggs Institute tool for cross‐sectional studies and quasi‐experimental studies [29] and, for measurement development studies, the COSMIN Risk of Bias Tool [30].

### 3.2 Data Synthesis

Due to the broad range of measures used that were identified, a narrative synthesis suitable for heterogeneous data was performed, guided by Popay et al. [74]. First, study data were categorised in terms of study design and characteristics. Second, outcome measure data were categorised under characteristics, psychometric properties and alignment with the modified Kirkpatrick framework [20]. Data were tabulated to compare and interpret the study design and measurement data. Comparison led to the identification of themes, which are reported below. To address the review question, findings were reported under three headings: (i) Study characteristics and designs used to measure outcome; (ii) Measure characteristics, organised by the concept being measured; and (iii) Alignment with the modified Kirkpatrick framework of evaluation. Established measures were reported under their original citation, with data on reliability and validity gathered from the study identified in the review or the cited study for the measure. Adapted measures were reported under the citation of their adapted version; only reliability and validity data for the adapted version were gathered.

## 4 Results

### 4.1 Study Characteristics and Designs

Nineteen studies met the criteria for this review. Fifteen studies were published between 2016 and 2023. Seven studies were conducted in Australia [31, 32, 42, 68, 69, 70, 71]. Most studies (*n* = 16) focused on a single profession, with only three studies including multiple groups of professionals [31, 33, 34]. Fourteen studies were conducted on undergraduate programmes; two included both undergraduate and postgraduate entry‐level training, one included only postgraduate, and one was a diploma‐level study. The service user involvement evaluated by the selected studies included the co‐production of an online teaching resource, sharing lived experience stories, co‐production and co‐delivery of simulation or teaching, and involvement in a workshop or student assessment. See Table 1 for the characteristics of the included studies.

**Table 1:** Study characteristics.

Authors	Year	Country	Study aims	Nature of service user involvement
Arblaster et al.	2023	Australia	To evaluate a multidisciplinary teaching resource on mental health recovery involving people with lived experience	Co‐production of an online teaching resource
Ferri et al.	2023	Italy	To evaluate an interprofessional education intervention in partnership with patient educators	Sharing lived experience stories
Feijoo‐Cid et al.	2022	Spain	To evaluate the impact of expert HIV patients providing teaching to Spanish nursing students	Co‐production and co‐delivery of teaching
Hache et al.	2022	France	To assess the impact of a workshop involving ‘patient teachers’ about patient‐focused communication	Co‐production and co‐delivery of teaching
Logan et al.	2022	Australia	To measure the impact of a face‐to‐face mental health service user‐led teaching initiative on levels of empathy in occupational therapy students	Co‐production and co‐delivery of teaching
Scanlan et al.	2022	Australia	To evaluate the outcomes achieved by students who were involved in a unit of study that was co‐designed and co‐delivered by service users	Co‐production and co‐delivery of teaching
Duffy et al.	2021	The United Kingdom	To compare the learning experience of social work students when they performed role‐plays with drama students, with when they worked with service users	Service user involvement in assessment
Hache et al.	2020	France	To measure the impact of a workshop involving ‘patient partners’ during a module focusing on patient education programmes (PEPs) in pharmacy training	Co‐production and co‐delivery of teaching
Ferri et al.	2019	Italy	To evaluate the effect of expert patient teaching on the development of empathy in nursing students	Sharing lived experience stories
Happell et al.	2019	Australia, Ireland, Finland, Norway, the Netherlands and Iceland	To evaluate the impact of expert by experience co‐produced nursing education and explore it as an internationally applicable principle for positively impacting student attitudes towards people with mental illness, consumer participation and recovery‐oriented nursing care	Co‐production and co‐delivery of teaching
Tobbell et al.	2018	The United Kingdom	To generate and validate a psychometric instrument which will allow educators to evaluate the new service user pedagogy	Involvement in developing the measure
Logan et al.	2018	Australia	To investigate if a service user involved in an oral assessment in a mental health unit impacted on engagement, anxiety states and academic performance in occupational therapy students	Service user involvement in assessment
Boukouvalas et al.	2018	Australia	To assess the impact of using people with a lived experience of mental illness as simulated patients on pharmacy students' attitudes towards and confidence in responding to suicidal ideation, following MHFA training	Co‐production and co‐delivery of simulation
Feijoo‐Cid et al.	2017	Spain	To evaluate nursing students' satisfaction with patients telling their lived experience stories—‘Expert Patient Illness Narratives’	Sharing lived experience stories
Cabiati and Raineri	2016	Italy	To assess the impact of a one‐day meeting between service users and carers with social work students	Service user involvement in the workshop
Byrne et al.	2014	Australia	To determine the degree of change in attitudes to service user involvement in the traditional taught module and compare it with attitude change in the recovery‐focused module involving service users	Co‐production and co‐delivery of teaching
Duffy, Das and Davidson	2013	The United Kingdom	To evaluate the involvement of service users in assessed role‐plays with students as part of their preparation for the practice learning module	Service user involvement in simulation
Bell et al.	2006	Australia	To assess the impact of participation by mental health consumers in pharmacy education	Sharing lived experience stories
Costello and Horne	2001	The United Kingdom	To evaluate a workshop involving the utilisation of patient teaching in a classroom setting	Sharing lived experience stories

Twelve studies applied mixed methods [31, 33, 35, 36, 37, 38, 39, 40, 41, 42]. The remaining seven studies used quantitative methods only. Study designs were mostly quasi‐experimental (*n* = 14), with three cross‐sectional studies and one randomised controlled trial. One study was a measure development study. Five studies included some form of comparison group [43, 68, 69, 71, 72] (see Table 2).

**Table 2:** Study design.

Study	Study design	Sample	Measures used	No. of participants (response rate)/Participant characteristics	Data collection	Data analysis
Arblaster et al. [31]	Mixed‐methods pre‐/post‐evaluation	Nursing and occupational therapy students	− *20‐Item Capabilities for Recovery‐Oriented Practice Questionnaire (CROP‐Q)* [44]	Pre‐test—56 student respondents (28 nurse/28 occupational therapy)	Survey shared through the university's learning management system, both before and after access to the online teaching resource.	Descriptive statistics. Significant differences were measured using the Wilcoxon signed‐rank test.
				49 female		
				Post‐test—28 student respondents (2 nurse/26 occupational therapy)		
				26 female		
Ferri et al. [33]	Mixed‐methods pre‐/post‐evaluation study	Undergraduate nursing, dietitian, speech therapy, medical, midwifery and OT students	− *Jefferson scale of Empathy—Health Professions Student* [45] − *Readiness for Interprofessional Learning Scale* (Italian version) [59]	292 students (94%)	Surveys were administered immediately before and after the workshop.	Descriptive statistics used to summarise demographics and survey scores. Wilcoxon signed‐rank test compared pre‐ and post‐test data for each item and statistical significance was established.
				13 OT, 15 dietetics, 15 midwifery, 126 medical and 133 nursing		
				222 female		
				Age range is 19–55; mean age 21 years with a standard deviation of 3.2 years		
Feijoo‐Cid et al. [38]	Mixed‐methods pre‐/post‐evaluation study	Undergraduate nursing students	− *Patient Practitioner Orientation Scale (PPOS)* [46] − *Knowledge attitudes and practices questionnaire on HIV/AIDS* (IPPF [47])	34 students (41%)	Survey was administered before the first workshop and after the last workshop.	Descriptive statistics, statistically significant difference measured.
				27 female		
				Age 21–36 (median 22)		
Hache et al. [40]	Mixed‐methods evaluative study	Undergraduate pharmacy students	− Student competency scoring	47 students (100%)	Patient and peer residents assessed learners' performance in a simulated interview against a purpose‐designed competency chart.	Mean and standard deviation, statistically significant difference measured and correlations calculated through Pearson's correlation coefficient.
				38 female/9 male		
				Age 22–25 years		
Logan et al. [71]	A quantitative quasi‐experimental two‐group comparison design study	Undergraduate occupational therapy students	− *Jefferson Scale of Empathy—Health Professions Student* [67]	138 students (64%)	Survey administered in online format via email, pre‐intervention survey administered a week before the session, and post‐intervention survey administered a week after.	Descriptive statistics and linear regression to assess the difference between groups at follow‐up, 95% confidence intervals and statistically significant difference measured. Confirmatory factor analysis was also carried out.
				Demographic data not collected		
Scanlan et al. [42]	Mixed‐methods pre‐/post‐evaluation study	Undergraduate occupational therapy students	− *Recovery Knowledge Inventory* [48] − *24‐Item Capabilities for Recovery‐Oriented Practice Questionnaire* (CROP‐Q) [42]	70 students (72%)	Paper‐based surveys were handed out at the beginning of class. Students who wanted to participate returned the surveys to a sealed returns box whilst teaching staff were out of the room.	Descriptive statistics, statistically significant difference measured.
				Age 19–30 years, average 20.7		
				59 female		
Duffy et al. [72]	Two‐group comparative study	Undergraduate and postgraduate social work students	− Purpose‐designed measure comparing their learning experiences with drama students and service user education	89 students (77%)	Online survey completed by each group after each intervention.	Descriptive statistics, statistically significant difference measured.
				77 female		
				Average age 29 years		
Hache et al. [39]	Mixed‐methods cross‐sectional evaluation study	Undergraduate pharmacy students	− Purpose‐designed measure seeking the student level of satisfaction, learning and transferability of learning	94 students (100%)	Surveys were administered at the end of the sessions.	Descriptive statistics, statistically significant difference measured.
				70 female		
				Age 20–23 years		
Ferri et al. [43]	Randomised control trial	Undergraduate nursing students	− *Balanced Emotional Empathy Scale* [49]	144 students (92%)	Two surveys were administered before and after the seminars.	Descriptive statistics, statistically significant difference measured.
				127 female		
			− *Jefferson Scale of Empathy—Health Professions Student* [45]	Aged 20.9 ± 2.6 (experimental group)/20.7 ± 2.6 (control group)		
Happell et al. [41]	Mixed‐methods pre‐/post‐evaluative survey	Undergraduate nursing students	− *Mental Health Nurse Education Survey* [50]	Total—193 students. Australia (*n* = 69), Ireland (*n* = 59) and Finland (*n* = 65)	Paper surveys were administered during classes before starting the module and soon after completion of the module.	Descriptive statistics and *t*‐tests were used to evaluate statistical significance in changes. Cronbach's *α* used to establish internal reliability. Principle component analysis, exploratory or confirmatory factor analysis used for relevant measure based on previous knowledge of psychometric properties of measure.
			− *Opening Minds Stigma Scale for Health Care Providers* [51]	160 female (84.7% Australia, 85.1% Ireland and 83.1% Finland)		
			− *Adapted Mental Health Consumer Participation Questionnaire* [50]	The majority aged between 18 and 29 (84.7% Australia, 86.6% Ireland and 83.1% Finland)		
Tobbell et al. [34]	Quantitative—measure development study	Undergraduate nursing, podiatry, midwifery, occupational therapy, ODP, physiotherapy and social work students	− *Huddersfield pedagogy scale* [34]	365 students across multiple professions at two universities	Online and paper copies of the survey were administered. Paper copies were administered during lectures.	*χ* ^2^ goodness‐of‐fit statistic, degrees of freedom, Comparative Fit Index, Tucker–Lewis Index, Root Mean Square Error of Approximation; all used for three alternative models of the scale (1 factor; 2 factor; bifactor [4 grouping factors]).
				299 female		
				Nursing = 150, podiatry = 50, midwifery = 46, OT = 27, ODP = 28, physiotherapy = 45 and social work = 19		
Logan et al. [32]	A quantitative quasi‐experimental study.	Undergraduate occupational therapy students	− *State form from the State‐Trait Anxiety Inventory for Adults* [52]	79 students (73%), 29 in control group, 33 in consumer and 17 unsure—Unsure group not included in planned analysis.	In the 24 h before completing the oral assessment, students completed the pre‐assessment survey. The post‐assessment survey was collected within 1 week of completing the oral assessment. It was distributed by a staff member outside of the teaching team, and panel members did not have access to the results until after the oral assessment.	Descriptive statistics used for sample characteristics, mean, standard deviation and 95% confidence intervals. Repeated‐measure ANOVA used to determine differences between the comparison and experimental groups. *t*‐test is used to determine the statistical difference between two groups.
			− Purpose‐designed measure—Visual analogue scales capturing students' perception of engagement based on time, energy and effort given.			
			− Student knowledge and understanding based on academic scoring			
Boukouvalas et al. [69]	Parallel group repeated‐measures study	Undergraduate and postgraduate pharmacy students	− *Adapted Attitudes Towards Suicide Scale* [53]	186 students (74%)	Data collection at three time points, immediately pre‐ and post‐MHFA and then at 2–4 weeks follow‐up.	Descriptive statistics, linear regression to determine the difference between groups, confidence intervals and statistical significance established.
				Median age 22 (Bpharm) and 24 (Mpharm)		
			− Questions asking about self‐perceived confidence in recognising, approaching and supporting people experiencing a suicide crisis	133 female		
Feijoo‐Cid et al. [37]	Mixed‐methods cross‐sectional evaluation study	Undergraduate nursing students	− Purpose‐designed measure seeking students' level of satisfaction	64 students (77%)	Survey was administered in the classroom after the final narrative.	Descriptive statistics. Statistically significant difference measured.
				50 female		
Cabiati and Raineri [54]	Quantitative—pre‐/post‐intervention study	Undergraduate social work students	− *Attitudes to Mental Illness Questionnaire (AMIQ)* [55]	100 students (not reported)	Online survey administered 2 weeks prior (pre‐test) and 2 weeks post meeting collected by an external researcher.	Descriptive analysis identifying change in pre/post score, reporting pre/post mean and pre/post standard deviation.
				90 female		
				Mean age 20 years		
Byrne et al. [70]	Quantitative—pre‐/post‐intervention study	Undergraduate nursing students	− *Adapted Mental Health Consumer Participation Questionnaire*	170 students (59%)	Participants responded to a survey at the start of the course and then repeated at the end of the course, both paper and online copies were distributed.	Descriptive statistics, principal component analysis. Statistically significant difference measured. Cohen's effect size statistic and Pearson's *r* for effect size. Missing data was replaced through Expectation Maximisation.
				Ages—18–39 years		
Duffy et al. [36]	Mixed‐methods pre‐/post‐evaluation study	Postgraduate social work students and teaching staff	− Purpose‐designed measure seeking views on students' experience of service user participation in assessment	8 staff (pre‐data collection only)	Pre‐assessment survey distributed at the start of the term, and the post‐assessment survey was collected directly after the role‐play.	Descriptive statistics
				50 students		
				46 female		
				Age 21–44 years, average age 28 years		
Bell et al. [68]	Two‐group, non‐randomised, comparative study.	Undergraduate pharmacy students	− *Adapted Attribution Questionnaire* [56]	211 students (92%)	Participants completed a baseline survey instrument before their involvement in the mental health lectures. The follow‐up survey was completed immediately after their involvement in the tutorial.	Descriptive statistics. Statistical analysis to compensate for missing data.
				Reported no significant difference between groups with respect to demographics, although figures were not provided.		
			− *Social distancing scale* [56]			
			− *Adapted survey on pharmacists' attitudes towards mental illness (no name*) [57, 58]			
			− Purpose‐designed measure—10 items related to professional service delivery by pharmacists			
Costello and Horne [35]	Mixed‐methods cross‐sectional evaluation study	Diploma‐level adult nursing students	− Purpose‐designed measure seeking students' level of satisfaction	67 students (97%). Age and gender not reported.	Paper survey distributed by a teacher not facilitating the main session at the end.	Descriptive statistics

All studies used convenience sampling based on the health professional education programme in which the study was taking place. It was not always possible to establish response rates because recruitment methods were not consistently reported. For 18 of the studies, the service user involvement activity was a mandatory part of the education programme, although the evaluation was optional. The only study that deviated from this approach was Arblaster et al. [31], where the online teaching resource was mandatory for occupational therapy students and optional for nursing students, explaining the lower response rate amongst the latter group. The study by Duffy et al. [72] is the only one that included staff in their sample. However, despite collecting data pre‐ and post‐service user involvement activity from students, only the pre‐activity data was collected from staff participants. The remaining studies included students as the sole sample group. No studies collected quantitative data from service users (see Table 2).

Data collection was either via an online survey or the distribution of paper surveys during class. Thirteen studies collected both pre‐ and post‐service user involvement activity data. Twelve of the studies measured the outcome of the service user involvement activity immediately [31, 32, 33, 36, 38, 39, 41, 42, 43, 54, 71]. Boukouvalas et al. [69] conducted repeated post‐activity data collection after the activity with a follow‐up 2–4 weeks later.

Ten studies used one outcome measure [31, 34, 35, 37, 39, 40, 54, 70, 71, 72], but across eight studies, up to four measures were used [32, 33, 38, 41, 42, 43, 68, 69]. All studies carried out descriptive analysis with the use of mean and standard deviation. Fourteen determined statistical significance [31, 32, 33, 37, 41, 42, 43, 69, 70, 71, 72], and three measured confidence intervals [32, 69, 71]. Two studies carried out statistical analysis to compensate for missing data [68, 70].

### 4.2 Measure Characteristics

Twenty‐nine measures were identified. Twelve measures were purpose‐designed [32, 35, 36, 37, 39, 40, 68, 69, 72], 13 were established measures and 6 were adaptations of established measures [42, 48, 68, 69, 70]. One publication [68] reported two separate adapted measures. Below, we first report the constructs, reliability and validity of all the measures, followed by the detailed characteristics of the purpose‐designed measures, the established measures and the adapted measures.

#### 4.2.1 Constructs Being Measured

There was no clear consensus regarding which constructs should measure the outcome of service user involvement; 12 constructs were identified across the measures. The most frequent construct measured was knowledge and attitudes on mental illness [42, 44, 48, 50, 51, 53, 55, 68, 69, 72, 73]. Other measures included students' attitudes towards service user involvement [41, 70, 72, student satisfaction [35, 36, 37], student empathy [49, 67], and students' academic performance [32, 40]. The full list of constructs measured is shown in Table 3.

**Table 3:** Characteristics of measures.

Measure	Cited study for measure	Citation for original measure (for adapted measures)	Construct being measured	Target population	No. of items	Structure (subscales)	Rating method
**Purpose‐designed measures**							
Student Competency Assessment	Hache et al. [40]		Academic Performance	Student Health Professional	14 items	− Building a relationship − Conducting a structured interview − Gathering information.	14 competency‐based skills assessed on a rating of 0 (non‐acquired) to 5 (mastered).
Purpose‐designed measure	Duffy et al. [72]	N/A	Attitude towards service user involvement	Student Health Professional	6 items	N/A	Likert scale 1 = extremely negative, 7 = extremely positive
Purpose‐designed measure	Hache et al. [39]	N/A	Student satisfaction, learning and transferability of the learning	Student Health Professional	Not reported	N/A	Likert—details not reported
Purpose‐designed measure	Boukouvalas et al. [69]	N/A	Knowledge and attitudes on mental illness	Student Health Professional	8 items	N/A	Likert scale—range not reported
Purpose‐designed measure—Visual analogue scales	Logan et al. [32]	N/A	Student engagement	Student Health Professional	6 items	N/A	10 cm anchored scales from 0 ‘no time/energy/effort’ through to 10 ‘great deal of time/energy/effort’
Academic grades	Logan et al. [32]		Academic Performance	Student Health Professional	1 item	N/A	Oral assessments marked using a rubric with grades ranging from 0 to 20
Purpose‐designed measure	Feijoo‐Cid et al [37]	N/A	Student satisfaction	Student Health Professional	34 items	N/A	Likert scale 1 = completely disagree, 5 = completely agree
Purpose‐designed measure	Duffy et al. [36]	N/A	Student satisfaction	Student Health Professional	Not reported	N/A	Likert scale 1 = extremely negative, 7 = extremely positive
Purpose‐designed measure	Bell et al. [68]	N/A	Knowledge and attitudes on mental illness	Student Health Professional	10 items	N/A	Likert scale 1 = strongly agree, 5 = strongly disagree
Purpose‐designed measure	Costello and Horne [35]	N/A	Student satisfaction	Student Health Professional	7 items	N/A	Mixture of 5‐point Likert scale, multiple choice and yes/no questions. Alongside qualitative questions
**Established measures**							
20‐Item Capabilities for Recovery‐Oriented Practice Questionnaire (CROP‐Q)	Arblaster [44]	N/A	Knowledge and attitudes on mental illness	Health Professionals	20 items	One total score in response to one of three vignettes	5‐point Likert scale. Respondents give a total score out of 100.
The Huddersfield Pedagogy Scale	Tobbell et al. [34]	N/A	Perceived presence of service users and Knowledge and attitudes on mental illness	Student Health Professionals	19 items	− Perceived presence of service users − Resultant learning	Likert scale 1 = strongly disagree, 5 = strongly agree
Readiness for Interprofessional Learning Scale (Italian version)	Sollami et al. [59]	N/A	Skills for interprofessional learning	Health Professionals	10 items	One total score	Likert scale 1 = strongly disagree, 5 = strongly agree
State form from the State‐Trait Anxiety Inventory for Adults	Spielberger et al. [52]	N/A	Anxiety	Tested across multiple population groups including students	20 items	N/A	Likert scale 1 = not at all, 4 = very much so
Opening minds stigma scale for healthcare providers	Modgill et al. [51]	N/A	Knowledge and attitudes on mental illness	Health Professionals	15 Items	− Social distancing attitudes to mental illness	Likert scale 1 = strongly disagree, 5 = strongly agree
						− Disclosure and help‐seeking	
Mental Health Consumer Participation Questionnaire	Happel et al. [50]	N/A	Attitudes towards service user involvement	Student Health Professionals	17 Items	− Consumer involvement − Lack of capacity − Sufficiency of services − Consumer academic − Consumer as staff	Likert scale 1 = strongly disagree, 7 = strongly agree
Mental Health Nurse Education Survey	Happell and Gough Nee Hayman‐White [50]	N/A	Knowledge and attitudes on mental illness	Student Health Professionals	10 items	− Negative stereotypes − Valuable contribution − Preparedness for the mental health field	Likert scale 1 = strongly disagree, 7 = strongly agree
Knowledge, attitudes and practices questionnaire on HIV/AIDS	International Planned Parenthood Federation [47]	N/A	Clinical knowledge and attitudes	Health Professionals	52 items	− Attitudes towards people living with HIV − HIV knowledge and practices	Range of true/false, multiple choice and Likert scale responses
Attitudes to Mental Illness Questionnaire (AMIQ)	Luty et al. [55]	N/A	Knowledge and attitudes on mental illness	Health Professionals	5 items	One total score in response to one of five vignettes	Likert scale—most likely (−2) to most unlikely (+2)
Jefferson Scale of Empathy—Health Professions Student	Hojat et al. [67]	N/A	Student empathy	Student Health Professional	20 items	− Perspective taking − Compassionate care − Standing in the patient's shoes	Likert scale 1 = strongly disagree, 7 = strongly agree.
Patient Practitioner Orientation Scale (PPOS)	Krupat et al. [46]	N/A	Perception of the patient–health professional relationship	Patients and physicians in GP surgeries	18 items	− Caring − Sharing	Likert scale 1 = strongly disagree, 6 = strongly agree
Balanced Emotional Empathy Scale	Mehrabian [49]	N/A	Student empathy	General Population	30 items	N/A	Items rated on a scale from −4 (very strong disagreement) to +4 (very strong agreement). 15 items rated positively, 15 rated negatively.
Social Distancing Scale (SDS)	Link et al. [73]	N/A	Knowledge and attitudes on mental illness	General Population	7 items	Provides an overall ‘Social Distancing Score'	Likert scale 1 = definitely willing, 4 = definitely unwilling
**Adapted measures from earlier studies**							
24‐Item Capabilities for Recovery‐Oriented Practice Questionnaire (CROP‐Q)	Scanlan et al. [42]	Arblaster [44]	Knowledge and attitudes on mental illness	Health Professionals	24 items	One total score in response to one of three vignettes	Likert scale 1 = strongly disagree, 4 = strongly agree
Adapted Attitudes Towards Suicide Scale	Boukouvalas et al. [69]	Renberg and Jacobsson [53]	Knowledge and attitudes on mental illness	General Population	21 items (of a 37‐item scale)	− Suicide as a right − Tabooing − Incomprehensibility − Situation Caused − Non‐communication	Likert Scale 1 = strongly agree, 5 = strongly disagree
Adapted Recovery Knowledge Inventory (RKI)	Happell, Byrne, and Platania‐Phung [48]	Bedregal et al. (2006)	Knowledge and attitudes on mental illness	Student Health Professionals	16 items (of a 20‐item scale)	− Roles of self‐definition and peers − Roles and responsibilities in recovery − Recovery as a process	Likert scale 1 = strongly disagree, 5 = strongly agree
Adapted Mental Health Consumer Participation Questionnaire	Byrne et al. [70]	Happell et al. [50]	Attitudes towards service user involvement	Student Health Professionals	24 items	− Consumer capacity − Consumer involvement − Consumer as staff	Likert scale 1 = strongly disagree, 7 = strongly agree
Adapted survey on pharmacists' attitudes towards mental illness (no name)	Bell et al. [68]	Cates et al. [58], Phokeo, Sproule and Raman‐Wilms [57]	Knowledge and attitudes on mental illness	Health Professional	16 items	N/A	Likert scale 1 = strongly agree, 5 = strongly disagree
Adapted Attribution Questionnaire	Bell et al. [68]	Corrigan et al. [56]	Knowledge and attitudes on mental illness	General Population	6 items from the Attribution Questionnaire (20 items)	− Frightened − Avoid	Likert scale 1 = strongly agree, 5 = strongly disagree

#### 4.2.2 Psychometric Properties of Measures

The Mental Health Nurse Education Survey [50], Capabilities for Recovery Oriented Practice Questionnaire (CROP‐Q) [44] and the Huddersfield Pedagogy Scale [34] were validated for the student health professional population. The Recovery Knowledge Inventory had been tested with student populations; however, the authors reported poor convergent validity and recommended further development of the measure [48]. The State‐Trait Anxiety Inventory has been extensively tested, demonstrating good reliability and validity across multiple population groups, including students [52, 60]. Four measures had tests of validation, however not with student health professional populations [49, 51, 55, 73]. Two measures had poor validity in their testing [46, 67]. Eight Measures had no validity reported [42, 47, 59, 61, 68, 69, 70].

Two of the 12 measures that tested for internal consistency measures had poor internal consistency (*α* < 0.7) [50, 69]. Four measures had internal consistency that varied across subscales [46, 51, 61, 70], three of which also varied based on geographical location [51, 61, 70], with the remaining seven receiving fair to good internal consistency [34, 49, 52, 59, 67, 73]. Three measures rated good test–retest reliability [49, 52, 55]. No other measures of reliability were identified. Full details on the reliability and validity of measures can be found in Table 4. Validity and reliability were not reported on any of the purpose‐designed measures and were therefore not included in the table.

**Table 4:** Psychometric properties of established and adapted measures.

Measure	Citation	Internal consistency	Test–retest reliability	Construct validity	Convergent validity	Content validity	Confirmatory factor analysis	Reliability	Validity
24‐Item Capabilities for Recovery‐Oriented Practice Questionnaire (CROP‐Q)	Scanlan et al. [42]	0	0	0	0	+	0	Not reported	Not reported
20‐Item Capabilities for Recovery‐Oriented Practice Questionnaire (CROP‐Q)	Arblaster [44]	0	0	0	0	+	0	Not reported	Content Validity in pilot testing—0.9–1.0
Adapted Attitudes Towards Suicide Scale	Boukouvalas et al. [69]	—	0	0	0	0	0	Poor internal consistency reported across the majority of the factors of the 10‐factor version *α* = 0.36–0.86	Developed in consultation with experts and ‘laymen’ supports face validity
Readiness for Interprofessional Learning Scale (Italian version)	Sollami et al. [59]	+	0	0	0	0	0	Reports good internal consistency *α* = 0.89–0.92	Not Reported
The Huddersfield Pedagogy Scale	Tobbell et al. [34]	+	0	0	0	0	+	Internal reliability using composite reliability for two‐factor model The perceived presence of service users (*α* = 0.89), resultant learning (*α* = 0.81)	Good validity through conventional confirmatory factor analysis and confirmatory bifactor modelling
Adapted Recovery Knowledge Inventory (RKI)	Happell et al. [48]	0	0	0	—	0	—	Exploratory factor analysis *α* = 0.75 for roles and responsibilities, *α* = 0.49 for roles of self‐definition and peers in recovery, *α* = 0.72 for recovery as a process	Poor convergent validity (loadings under 0.50) Poor factor loadings throughout
Adapted Mental Health Consumer Participation Questionnaire	Byrne et al. [70]	+	0	0	0	0	0	Good internal consistency using Cronbach's *α* in all scales apart from consumer capacity *α* = 0.68–0.86 (consumer capacity), *α* = 0.78–0.94 (consumer involvement) and 0.73–0.80 (consumer as staff)	Not reported
Opening Minds Stigma Scale for Health Care Providers	Modgill et al. [51]	—	0	+	0	0	0	Internal consistency range across study locations using Cronbach's	States good construct validity (although details not provided)
								*α* = 0.60–0.76 (social distancing), *α* = 0.39–0.79 (attitudes to mental illness) and *α* = 0.5–0.67 (disclosure and help‐seeking)	
Mental Health Consumer Participation Questionnaire	Happell et al. [50]	—	0	0	0	0	0	Internal consistency range across study locations using Cronbach's *α* = 0.72–0.9 (Consumer Involvement), *α* = 0.55–0.74 (Lack of Capacity), *α* = 0.58–0.74 (Sufficiency of services), *α* = 0.54–0.85 (Consumer Academic), *α* = 0.64–0.8 (Consumer as *α* = Staff)	Good face validity reported, but no details provided
Mental Health Nurse Education Survey	Happell and Gough Nee Hayman‐White [50]	—	0	+	0	+	0	Internal consistency range across study locations using Cronbach's *α* = 0.7–0.88 (negative stereotypes), *α* = 0.52–0.79 (valuable contribution), *α* = 0.76–0.84 (preparedness for mental health field). Scores vary based on the country of the respondents.	Good content validity based on expert opinion States good construct validity (although details not provided)
Knowledge, attitudes and practices questionnaire on HIV/AIDS	IPPF International Planned Parenthood Federation [47]	0	0	0	0	0	0	Not reported	Not reported
Adapted survey on pharmacists' attitudes towards mental illness (no name)	Bell et al. [68]	0	0	0	0	0	0	Not Reported	Not Reported
Adapted Attribution Questionnaire	Bell et al. [68]	0	0	0	0	0	0	Not reported	Not reported
Attitudes to Mental Illness Questionnaire (AMIQ)	Luty et al. [55]	0	+	+	0	0	0	Good test–retest reliability and alternative test reliability	Good construct and criterion validity
Jefferson Scale of Empathy—Health Professions Student	Hojat et al. [67]	+	0	0	0	0	—	Reports internal consistency *α* = 0.85 [43]	Poor fit confirmatory factor analysis observed using RMSEA and CFI [71]
Patient Practitioner Orientation Scale (PPOS)	Krupat et al. [46]	—	0	—	0	0	—	Internal consistency using Cronbach's *α* (physicians: *α* = 0.73, 0.67 and 0.52, respectively, for Total, sharing and caring; for patients *α* = 0.79, 0.72 and 0.52)	Poor factor loadings throughout (0.1–0.3) Poor construct validity of the caring subscale through correlation with the context‐specific scale.
Balanced Emotional Empathy Scale	Mehrabian [49]	+	+	+	0	0	0	Good internal consistency *α* = 0.87	Good construct validity with the emotional empathy tendency scale (correlation of 0.77) Good test–retest reliability *R* = 0.77
Social Distancing Scale (SDS)	Link et al. [73]	+	0	0	0	0	0	Good internal consistency using Cronbach's *α* = 0.86	Previous studies conducted on general population; no tests of validation conducted on this sample.
State form from the State‐Trait Anxiety Inventory for Adults	Spielberger et al. [52]	+	+	+	0	0	0	Good internal consistency *α* = 0.86 [60]	Good construct validity [60]

*Note:* Rating: + = Positive, — = Negative, 0 = no information available.

#### 4.2.3 Purpose Designed Measures

Ten out of the 12 purpose‐designed measures were self‐administered questionnaires [32, 35, 36, 37, 39, 40, 68, 69, 72]. Of these, six used Likert scales as their data collection method [36, 37, 39, 68, 69, 72], and one also asked categorical questions [35]. One measure consisted of anchored scales [32]. Hache et al. [39] used a purpose‐designed tool to measure relevance, learning and achievement transfer. The authors referenced the Kirkpatrick framework [62] but offered little detail on its application. In their study, one questionnaire was broken into two parts, the first measured relevance by asking students to self‐rate satisfaction, level of learning and transferability. The second part asked students to measure their perceived knowledge of pre‐defined learning outcomes.

Academic performance was measured in the studies by Logan et al. [32] and Hache et al. [40]. Logan et al. [32] used a marking rubric to assess the knowledge and understanding demonstrated by the student during the assessment. Student performance was measured in the study by Hache et al. [40], which aimed to understand the impact of service user involvement in student role‐play assessment. Hache et al. [40] used a competency‐based assessment specifically designed to assess student performance during a role‐play. In both cases, measures were completed by assessors, who in Logan et al. [32] included service users.

#### 4.2.4 Established Measures Utilised in Earlier Studies

The 13 established measures were all self‐administered questionnaires involving Likert scales in response to a statement. One measure, the knowledge, attitudes and practices questionnaire on HIV/AIDS, included a range of categorical responses in addition to the Likert scale [47]. Twelve of the measures were broken down into subscales [34, 41, 46, 47, 48, 50, 51, 52, 67, 68, 69]. Two measures provide an overall score from the measure [49, 59]. The Capabilities for Recovery‐Oriented Practice Questionnaire [44] and Attitudes to Mental Illness Questionnaire [55] gathered responses to vignettes and provided one overall score, with higher scores indicating more positive associations.

#### 4.2.5 Adapted Measures From Earlier Studies

Six adapted measures were identified. These were all self‐administered questionnaires involving Likert scales in response to a statement [42, 48, 68, 69, 70]. Four measures were broken down into subscales [48, 68, 69, 70]. The adapted version of the Capabilities for Recovery‐Oriented Practice Questionnaire provided an overall score in response to one of three vignettes [42]. Adaptions made to the measures included reducing the number of items [68, 69] or using an earlier version of an established measure [42, 70]. The Adapted Recovery Knowledge Inventory was adapted by reducing the number of items to 16 from a 20‐item scale, reducing the Likert scale to a 5‐point measure and removing the ‘not sure’ option after testing with a student population [48]. Except for the Recovery Knowledge Inventory, there was no theoretical underpinning for why any adaptation was made.

### 4.3 Extent to Which the Measures Align With the Modified Kirkpatrick Framework of Evaluation

Measures aligned with Levels 1–3 of the modified Kirkpatrick's framework [20]. Sixteen of the measures aligned with Level 2a (modification of attitudes/perceptions) of the framework. The remainder of measures aligned with Level 1 (learner's reaction) (*n* = 9), Level 2b (acquisition of knowledge/skills) (*n* = 6) and Level 3 (change in behaviour) (*n* = 2). Three measures aligned with two levels of the framework; however, none of the measures, or combinations of measures, used in the selected studies aligned with more than two levels. None of the measures identified in this review aligned with Levels 4a (change in organisational practice) and 4b (benefits to patients) of the framework. Full details of measure alignment with the modified Kirkpatrick Framework can be found in Table 5.

**Table 5:** Alignment with the modified Kirkpatrick framework of evaluation [20].

Measure (author, year of publication)	Level 1—Student reaction	Level 2a—Modification of attitudes and perceptions	Level 2b—Acquisition of knowledge and skills	Level 3—Behaviour change	Level 4a—Change in organisational practice	Level 4b—Benefit to patient
**Purpose‐Designed Measures**						
Academic Grades [32]			X			
Student Competency Assessment [40]				X		
Duffy et al. [72]	X					
Hache et al. [39]	X	X				
Boukouvalas et al. [69]		X				
Logan et al. [32]				X		
Feijoo‐Cid et al. [37]	X					
Duffy et al. [36]	X					
Bell et al. [68]		X				
Costello, Horne [35]	X					
**Established Measures**						
24‐Item Capabilities for Recovery‐Oriented Practice Questionnaire (CROP‐Q) [42]			X			
20‐Item Capabilities for Recovery‐Oriented Practice Questionnaire (CROP‐Q) [44]			X			
Adapted Attitudes Towards Suicide Scale [69]		X				
Readiness for Interprofessional Learning Scale (Italian version) [59]			X			
The Huddersfield Pedagogy Scale [34]	X	X				
Recovery Knowledge Inventory (RKI) [48]			X			
Adapted Mental Health Consumer Participation Questionnaire [70]		X				
Opening Minds Stigma Scale for Health Care Providers [51]		X				
Mental Health Consumer Participation Questionnaire [50]		X				
Mental Health Nurse Education Survey [50]		X				
Knowledge, attitudes and practices questionnaire on HIV/AIDS [47]		X	X			
Adapted Attribution Questionnaire [68]	X					
Adapted survey on pharmacists' attitudes towards mental illness (no name) [68]		X				
Attitudes to Mental Illness Questionnaire (AMIQ) [55]		X				
Jefferson Scale of Empathy—Health Professions Student [67]		X				
Patient Practitioner Orientation Scale (PPOS) [46]	X					
Balanced Emotional Empathy Scale [49]		X				
Social Distancing Scale [73]		X				
State form from the State‐Trait Anxiety Inventory for Adults [52]	X					

## 5 Discussion

The aim of this study was to describe the study designs and outcome measures used to evaluate service user involvement in non‐medical health professionals' entry‐level education. Most studies focused on outcomes for students and were small‐scale quasi‐experimental evaluations of a single teaching activity involving pre‐post data collection. The majority of the measures were purpose‐designed or adapted from published measures and not validated for measuring service user involvement. None of the measures aligned with Level 4 (organisational results) of the Kirkpatrick framework.

Studies were often small‐scale quasi‐experimental evaluations of a workshop or teaching activity involving service users restricted to one university programme. Whilst this limits generalisability to the health professional student population, there is potential for transferability to other similar settings. Collecting data before and after service user activity was a common practice among the studies reviewed. The majority collected data immediately after the activity, a method that supported a high response rate and captured students' perspectives. In contrast, studies that implemented a longer delay before data collection reported lower response rates. Notably, Boukouvalas et al. [69] was the only longitudinal study included in this review, reinforcing findings from other reviews on service user involvement, which highlight the scarcity of evidence on long‐term outcomes [63]. Samples included in the studies focused on student health professionals. Whilst one study included educators in the data collection, they were only included in the pre‐service user activity data collection, and little detail was provided on their results [36]. No studies included the service users as participants in their quantitative data collection. However, the study by Hache et al. [40] gathered student assessment data from the perspective of the service users; this may be interpreted as patient satisfaction in learning and provides the external validation of the learning effect. Other reviews have identified that service users report personal, cultural and organisational impact as a result of their involvement [64]. It is therefore important that the potential effect of the involvement is considered from the service user's perspective. Multi‐site studies utilising comparison groups can build on the existing evidence, improving applicability to the student health professional population and further demonstrating the influence of service user involvement.

This review identified a range of purpose‐designed and established measures. The established measures identified were often developed for another context other than specifically measuring the impact of service user involvement in education. With several established measures adapted from their original form, often with little explanation or theoretical underpinning, adaptations were made to meet the needs of the study, or when measures are in their infancy and require refining. This diverse range of measures resulted in a broad range of constructs being measured. Only the Mental Health Consumer Participation Questionnaire (MHCPQ) [61, 70] and the Huddersfield Pedagogy Scale [34] specifically referenced service user involvement in their scale; however, both measures require further refinement. The Jefferson Scale of Empathy—Health Professions Student [33, 43, 71], MHCPQ [41, 70] and CROP‐Q [31, 42] were used across multiple studies. However, different versions of the MHCPQ and CROP‐Q were applied in their respective studies. The diversity in measures used reflects the broad range in the application of service user involvement, as well as the different perspectives on the benefits of service user involvement. Whilst topic‐specific measures are a valid way of measuring change in attitude or knowledge towards a topic, without a comparison group, they do not provide clear evidence that any change is a result of service user involvement [65]. Further development and validation of measures on service user involvement is needed.

The variation in measures and the tests of reliability/validity used created challenges in directly comparing each measure. Psychometric data around the reliability and validity of the measures was either lacking or incomplete in many studies, and there was a dependence on using reliability and validity data from previous studies, irrespective of the context in which the measure was previously used. When measures had been adapted in some form, it is assumed by the authors that they would still hold the same psychometric properties as the originals. Validity was demonstrated in a variety of ways; however, the samples on which the measures were validated did not always share the same characteristics as those on which they were applied. The high number of purpose‐designed measures used for a single study further contributes to measures that have limited evidence of reliability and validity. These measures are typically designed to meet the needs of the study with no evidence of a supportive theory. There is a need for further multi‐site research utilising larger samples to enable testing of the validity and reliability of the measures used in the identified studies, as well as to gain the perspective of service users and educators.

All the measures identified in this review aligned with Levels 1–3 of the modified Kirkpatrick Framework [20]; none aligned with Level 4. These results are similar to other reviews using Kirkpatrick's framework that highlight the complexities in measuring tangible organisational impact as a result of a pedagogical initiative [66]. Measures aligning with Levels 2a and 2b demonstrate that learning has taken place; however, the self‐administered nature of the measures leaves them open to bias, where students might overstate their learning. Measurement through academic grades and competencies can provide some objectivity at these levels by having a third party, such as an educator, administer the measure. None of the measures aligned with all four of the levels, indicating a disconnect between health professional education and the impact on practice. Whilst the framework provides a useful structured approach to evaluation, evaluating all levels can be complex, resource‐intensive and time‐consuming, particularly evaluating behaviour and results, which require a longer time frame for data collection, pushing studies to restrict their evaluations to the first two levels.

### 5.1 Limitations of Review

This review may be limited by the inclusion of primary peer‐reviewed articles, which removed other sources such as books and grey literature. Since this review only explored measures applied in non‐medical health professionals' entry‐level education, it does not report on measures used in psychology or medical training. Including these additional professions in this review would have introduced substantial heterogeneity in terms of educational models, expectations and evaluation approaches, which would have compromised the ability to meaningfully synthesise findings or make valid comparisons. Our review provides insight as to where guidance for curriculum developers and policymakers is needed. This review identified studies that have involved service users in the teaching, assessment and curriculum development on health professional entry‐level education programmes. Service user involvement can also include a range of activities, including recruitment of students and university staff, strategic decision‐making and organisational planning, which were not identified from the literature search. The authors recognise that this review explores just a small element of service user involvement, cultural shifts in the way organisations work, which cannot always be measured in terms of impact.

## 6 Conclusion

This review highlights significant gaps in the evaluation of service user involvement in non‐medical health professionals' entry‐level education. Most studies employed small‐scale, quasi‐experimental designs focused on student outcomes. The reliance on non‐validated, purpose‐designed measures and the broad range of constructs measured limits the generalisability and comparability of the findings of the selected studies. The absence of measures aligning with Level 4 of the Kirkpatrick Framework reflects the challenges in assessing long‐term organisational impact. The exclusion of service users' and educators' perspectives in quantitative evaluations represents a missed opportunity to capture the broader effects of their involvement. For the ambitions of service user involvement to move beyond policy compliance towards genuine partnership, educators and researchers must invest in the development of robust, stakeholder‐informed outcome measures. Such tools are essential not only for curriculum improvement but also for accountability to the people whose lived experience shapes professional learning. Future research should not only prioritise multi‐site studies with validated measures but also look to further understand the desired outcomes of service user involvement, incorporating service user and educator perspectives, to enhance the robustness and applicability of findings.

## Author Contributions

**Jonathan Green:** conceptualisation, investigation, writing – original draft, methodology, writing – review and editing, formal analysis, project administration. **Jennifer Oates:** conceptualisation, writing – review and editing, methodology, supervision. **Sam Robertson:** conceptualisation, writing – review and editing, supervision, methodology. **Elizabeth Barley:** conceptualisation, writing – review and editing, methodology, supervision. **Chris Jacobs:** conceptualisation, investigation, writing – original draft, validation, methodology, writing – review and editing, supervision.

## Ethics Statement

The authors have nothing to report.

## Conflicts of Interest

The authors declare no conflicts of interest.

## Supporting information

Inclusion/Exclusion Criteria.

Search Strategy.

## Data Availability

The data that support the findings of this study are available in the tables of this article.
