# Supplementary material for: Evaluating the Study Designs and Outcome Measures Used in Service User Involvement in Health Professional Entry‐Level Education: A Systematic Review
Source: Health Expect. 2025 Sep 27;28(5):e70439. doi: 10.1111/hex.70439 (PMC12476030; doi:10.1111/hex.70439)
Supplement: Supplementary file 1 — Inclusion/Exclusion Criteria. [file HEX-28-e70439-s001.docx]

| Inclusion/Exclusion Criteria |  |
| --- | --- |
| Inclusion | Exclusion |
| Peer reviewed studies in academic journals | Studies published in any other format |
| Original Research | Study that is not reporting on original research |
| Published between the year 2000-2024 | Studies published prior to the year 2000 |
| Studies written in the English language | Studies published in any other language |
| Studies are conducted within the University setting | Studies conducted in any other setting |
| Studies relating to the impact of service user involvement in the design, teaching or assessment of students in pre-registration training of nurses, midwives, social workers, pharmacists and allied health professionals*. | Studies related to service user involvement in any other profession, or in post-registration training and education, or that describe the nature of service user involvement rather than measuring the impact of involvement. |
| Studies that use a quantitative methodology as all off, or part of their data collection and analysis. | Studies that use only qualitative methodology as their data collection and analysis. |
|  | Studies where you can not differentiate nurses, midwives, social workers, pharmacists and allied health professionals participants from other participant groups |

AHP List - Art therapists, Dietitians, Drama therapists, Music therapists, Occupational therapists, Operating department practitioners, Orthoptists, Osteopaths, Paramedics, Physiotherapists, Podiatrists, Prosthetists and orthotists, Radiographers, Speech and language therapists
