# Supplementary material for: Evaluating the Study Designs and Outcome Measures Used in Service User Involvement in Health Professional Entry‐Level Education: A Systematic Review
Source: Health Expect. 2025 Sep 27;28(5):e70439. doi: 10.1111/hex.70439 (PMC12476030; doi:10.1111/hex.70439)
Supplement: Supplementary file 2 — Search Strategy. [file HEX-28-e70439-s002.docx]

| CINAHL & MEDLINE | |
| --- | --- |
| 1 | AB ( student* OR nurs* OR midwif* OR “social work*” OR “occupational therap*” OR "art therap*" OR "drama therap*" OR "music therap*" OR podiat* OR dietitian* OR "operating department practitioner*" OR orthopist* OR osteopath* OR paramedic* OR physiotherapist* OR prosthetist* OR orthotist* OR radiograph* OR "speech and language therapist*" OR ahp OR Pharmacist) OR TI ( student* OR nurs* OR midwif* OR “social work*” OR “occupational therap*” OR "art therap*" OR "drama therap*" OR "music therap*" OR podiat* OR dietitian* OR "operating department practitioner*" OR orthopist* OR osteopath* OR paramedic* OR physiotherapist* OR prosthetist* OR orthotist* OR radiograph* OR "speech and language therapist*" OR ahp OR Pharmacist) |
| 2 | MH (“Nurses+” OR "OR “students, nursing” Midwifery" OR “Social Workers” OR “occupational therapists” OR “Art Therapy” OR “psychodrama” OR “music therapy” OR “Podiatry” OR “nutritionist” OR “orthoptics” OR Osteopathic Physicians" or “paramedics” or “physical therapists” or “speech therapy” or “language therapy” OR “allied health personnel” OR “Pharmacist” |
| 3 | S1 or S2 |
| 4 | AB ( "service user*" OR patient* OR carer* OR "lived experience*" OR "expert by experience" OR “public advisor*” OR “Consumer*” ) OR TI ("service user*" OR patient* OR carer* OR "lived experience*" OR "expert by experience" OR “public advisor*” OR “Consumer*” ) |
| 5 | MH (“patients+”) |
| 6 | S4 OR S5 |
| 7 | AB ( (education OR learning OR teach* OR assessment) ) OR TI ( (education OR learning OR teach* OR Assessment) ) |
| 8 | MH (“education+) |
| 9 | S7 OR S8 |
| 10 | AB ( (involve* OR participat* OR ppi OR ppie OR Co-production OR coproduction) ) OR TI ( (involve* OR participat* OR ppi OR ppie OR Co- production OR coproduction) ) |
| 11 | MH (“patient participation”) |
| 12 | S10 OR S11 |
| 13 | S3 AND S6 AND S9 AND S12 |
|  | Limiters –  Publication  Date: 20000101- 20241231.  Peer Reviewed  Narrow by Language: - English |

| APA Psych Info | |
| --- | --- |
| 1 | TI ( student* OR nurs* OR midwif* OR “social work*” OR “occupational therap*” OR "art therap*" OR "drama therap*" OR "music therap*" OR podiat* OR dietitian* OR "operating department practitioner*" OR orthopist* OR osteopath* OR paramedic* OR physiotherapist* OR prosthetist* OR orthotist* OR radiograph* OR "speech and language therapist*" OR ahp OR Pharmacist ) OR AB ( student* OR nurs* OR midwif* OR “social work*” OR “occupational therap*” OR "art therap*" OR "drama therap*" OR "music therap*" OR podiat* OR dietitian* OR "operating department practitioner*" OR orthopist* OR osteopath* OR paramedic* OR physiotherapist* OR  prosthetist* OR orthotist* OR radiograph* OR "speech and language therapist*" OR ahp OR Pharmacist) |
| 2 | DE "Nurses" OR DE "Nurse Practitioners" OR DE "Psychiatric Nurses" OR DE "Public Health Service Nurses" OR DE "School Nurses" OR DE “Allied health Personnel” OR DE “Midwifery” OR DE “Pharmacists” |
| 3 | S1 or S2 |
| 4 | TI ( "service user*" OR patient* OR carer* OR "lived experience*" OR "expert by experience" OR “public advisor*” OR “Consumer*” ) OR AB ( "service user*" OR patient* OR carer* OR "lived experience*" OR "expert by experience" OR “public advisor*” OR “Consumer*” ) |
| 5 | DE "Patients" |
| 6 | S4 OR S5 |
| 7 | TI ( (education OR learning OR teach* OR assessment) ) OR AB ( (education OR learning OR teach* OR Assessment) ) |
| 8 | DE "Education" |
| 9 | S7 OR S8 |
| 10 | TI ( (involve* OR participat* OR ppi OR ppie OR Co-production OR coproduction) ) OR AB ( (involve* OR  participat* OR ppi OR ppie OR Co-production OR coproduction) ) |
| 11 | DE “Involvement” |
| 12 | S10 OR S11 |
| 13 | S3 AND S6 AND S9 AND S12 |
|  | Limiters - Publication  Year: 2000-2024  Narrow by Language: - English |
